# Supplementary material for: Continuous quality improvement with a two-step strategy effective for mass SARS-CoV-2 screening at the Tokyo 2020 Olympic and Paralympic Games
Source: PLoS One. 2024 Sep 26;19(9):e0304747. doi: 10.1371/journal.pone.0304747 (PMC11426491; doi:10.1371/journal.pone.0304747)
Supplement: Attachment — Submitted filename: Response_to_Reviewers.docx [file pone.0304747.s001.docx]

**Continuous quality improvement with a two-step strategy effective for mass SARS-CoV-2 screening at the Tokyo 2020 Olympic and Paralympic Games**

**Manuscript ID:** PONE-D-24-17763

**Response to Reviewer 1**

Thank you for reviewing our manuscript and for providing comments, which have helped us to considerably improve our manuscript. We have made revisions based on your comments and have provided our responses below. We hope that our responses and revisions adequately address your comments.

Reviewer #1: This manuscript is interesting in the field of laboratory medicine. However, the purpose is obscure and completely retrospective fashion. I can not find the novel point in this manuscript. In addition, the conclusion is not new for the researcher engaged in the field.

**Response**: We have revised the manuscript to highlight the novelty and purpose more clearly.

We revised the Introduction so that readers can understand the purpose and the novelty more clearly, as follows:

**(Lines 67-85)**

“The scientific framework this strategy has been reported previously. This strategic allocation of PCR tests to cases that were CLEIA-positive or inconclusive not only maintained accuracy but also significantly improved logistical efficiency in administering mass screening. To detect the virus with sufficient time to contain the spread as well as to maintain their daily training activities prior to competing in events and facilitate the timely arrival of athletes at their game venues, all participating athletes and others staying in the Olympic Village were screened daily for SARS-CoV-2 infection with this two-step strategy. Although this strategy had been confirmed to work in a short period for screening travellers at international airports, its utility for mass-gathering sporting events had not previously been evaluated. We implemented this strategy in an international multi-sports event, i.e. the Tokyo 2020 Olympic and Paralympic Games, implementing daily screening and longitudinal follow-up of all athletes.

The integration of a testing system with an information management system covering all steps from specimen collection to reporting of the results, enabled confirmatory test results to be provided within 12 hours, aiming for a mean TAT of 150 minutes for confirmatory tests. In addition, our challenge was to avoid the risk of infection when gathering at the collection booth; therefore, we adopted a policy of self-collection of saliva in individuals’ own rooms in their living quarters. However, self-sampling in individual rooms posed a risk of poor specimen quality due to suboptimal preparation, collection, and storage.”

1. The scientific framework of this manuscript has been described in the previous studies with a prospective study, showing a two-step strategy for detection of SARS-CoV-2 infection comprising screening of self-collected saliva samples using a chemiluminescence enzyme immunoassay (CLEIA) followed by confirmatory testing of saliva and nasopharyngeal swab (NPS) samples using polymerase chain reaction (PCR). Self-collected saliva enables simultaneous parallel sample collection and rapid processing, which are in need at sites of mass screening. Although this strategy had been confirmed to work in a short period for screening travellers at international airports, its utility for mass-gathering sporting events had not previously been evaluated. This manuscript described about implementation of this two-step strategy for detection of SARS-CoV-2 infection using self-collected saliva in an international multi-sports event, i.e. Tokyo 2020 Olympic and Paralympic Games. Our challenge was to build-up of daily screening and longitudinal follow-up of a large number of athletes and official staffs up to 14,000 per day during the participation at the Games, in order to contain the infection.

2. To detect SARS-CoV-2 infections as early as possible and to take measures to prevent further spread, the two-step strategy was adopted to meet the mass screening requirements of the event. Our challenge was to ensure that all athletes were able to maintain their daily training activity prior to competing in events and facilitate timely arrival of athletes at the game venue. The integration of a testing system with an information management system covering all steps from specimen collection to reporting of the results enabled confirmatory test results to be provided within 12 hours, with the target of a mean turnaround time (TAT) of 150 minutes for confirmatory tests.

3. Tests with self-collected saliva enables simultaneous parallel sample collection and rapid processing, which has been shown to be useful for mass screening of travelers at airports. In the Tokyo Olympic and Paralympic Games, our challenge was to avoid the risk of infection when gathering at the collection booth; therefore, we adopted a policy of self-collection of saliva in individuals’ own rooms in their living quarters. However, self-sampling in individual rooms posed a risk of poor specimen quality due to suboptimal preparation, collection, and storage. To ensure the accuracy of the COVID-19 screening test, we implemented continuous quality improvement through rigorous quality assurance measures, performance/specimen quality monitoring by an internal quality assessment, and a chronological chart analysis for individual cases.

These challenges were addressed for quality improvement on a real-time basis during the Games. The continuous quality improvement and the two-step strategy for mass COVID-19 screening contributed to the success of international major sporting events during the pandemic. The quality practice, systems, and workflows of testing with continuous quality improvement, described in this report could serve as a model for future mass-gathering sporting events during similar major infectious disease epidemics.

**Continuous quality improvement with a two-step strategy effective for mass SARS-CoV-2 screening at the Tokyo 2020 Olympic and Paralympic Games**

**Manuscript ID:** PONE-D-24-17763

**Response to Reviewer 2**

Thank you for reviewing our manuscript and for providing comments, which have helped us to considerably improve our manuscript. We have made revisions based on your comments and have provided our responses below. We hope that our responses and revisions adequately address your comments.

Reviewer #2: In this study, Miyachi et al. described a process of two-step strategy for large-scale screening of SARS-CoV-2 at the Tokyo 2020 Olympic. Although this paper is submitted as a research article, almost no data presented. Important studies that are the scientific backbone of this airport quarantine system using saliva samples are missing. They should be introduced with comments and references (Yokota, Clin Infect Dis 2021: Yokota, Lancet Microbes 2021).

**Response**: As suggested, we have cited references to the important studies that are the scientific backbone of the airport quarantine system using saliva samples (Yokota, Clin Infect Dis 2021: Yokota, Lancet Microbes 2021).

We summarized the points addressed in this manuscript, as shown below. Accordingly, description in the introduction and the discussion was changed so that the readers can understand them, as follows:

**(Lines 67-85)**

“The scientific framework this strategy has been reported previously. This strategic allocation of PCR tests to cases that were CLEIA-positive or inconclusive not only maintained accuracy but also significantly improved logistical efficiency in administering mass screening. To detect the virus with sufficient time to contain the spread as well as to maintain their daily training activities prior to competing in events and facilitate the timely arrival of athletes at their game venues, all participating athletes and others staying in the Olympic Village were screened daily for SARS-CoV-2 infection with this two-step strategy. Although this strategy had been confirmed to work in a short period for screening travellers at international airports, its utility for mass-gathering sporting events had not previously been evaluated. We implemented this strategy in an international multi-sports event, i.e. the Tokyo 2020 Olympic and Paralympic Games, implementing daily screening and longitudinal follow-up of all athletes.

The integration of a testing system with an information management system covering all steps from specimen collection to reporting of the results, enabled confirmatory test results to be provided within 12 hours, aiming for a mean TAT of 150 minutes for confirmatory tests. In addition, our challenge was to avoid the risk of infection when gathering at the collection booth; therefore, we adopted a policy of self-collection of saliva in individuals’ own rooms in their living quarters. However, self-sampling in individual rooms posed a risk of poor specimen quality due to suboptimal preparation, collection, and storage.”

1. The scientific framework of this manuscript has been described in the previous studies with a prospective study, showing a two-step strategy for detection of SARS-CoV-2 infection comprising screening of self-collected saliva samples using a chemiluminescence enzyme immunoassay (CLEIA) followed by confirmatory testing of saliva and nasopharyngeal swab (NPS) samples using polymerase chain reaction (PCR). Self-collected saliva enables simultaneous parallel sample collection and rapid processing, which are in need at sites of mass screening. Although this strategy had been confirmed to work in a short period for screening travellers at international airports, its utility for mass-gathering sporting events had not previously been evaluated. This manuscript described about implementation of this two-step strategy for detection of SARS-CoV-2 infection using self-collected saliva in an international multi-sports event, i.e. Tokyo 2020 Olympic and Paralympic Games. Our challenge was to build-up of daily screening and longitudinal follow-up of a large number of athletes and official staffs up to 14,000 per day during the participation at the Games, in order to contain the infection.

2. To detect SARS-CoV-2 infections as early as possible and to take measures to prevent further spread, the two-step strategy was adopted to meet the mass screening requirements of the event. Our challenge was to ensure that all athletes were able to maintain their daily training activity prior to competing in events and facilitate timely arrival of athletes at the game venue. The integration of a testing system with an information management system covering all steps from specimen collection to reporting of the results enabled confirmatory test results to be provided within 12 hours, with the target of a mean turnaround time (TAT) of 150 minutes for confirmatory tests.

3. Tests with self-collected saliva enables simultaneous parallel sample collection and rapid processing, which has been shown to be useful for mass screening of travelers at airports. In the Tokyo Olympic and Paralympic Games, our challenge was to avoid the risk of infection when gathering at the collection booth; therefore, we adopted a policy of self-collection of saliva in individuals’ own rooms in their living quarters. However, self-sampling in individual rooms posed a risk of poor specimen quality due to suboptimal preparation, collection, and storage. To ensure the accuracy of the COVID-19 screening test, we implemented continuous quality improvement through rigorous quality assurance measures, performance/specimen quality monitoring by an internal quality assessment, and a chronological chart analysis for individual cases.

These challenges were addressed for quality improvement on a real-time basis during the Games. The continuous quality improvement and the two-step strategy for mass COVID-19 screening contributed to the success of international major sporting events during the pandemic. The quality practice, systems, and workflows of testing with continuous quality improvement, described in this report could serve as a model for future mass-gathering sporting events during similar major infectious disease epidemics.

**Continuous quality improvement with a two-step strategy effective for mass SARS-CoV-2 screening at the Tokyo 2020 Olympic and Paralympic Games**

**Manuscript ID:** PONE-D-24-17763

**Response to Reviewer 3**

Reviewer #3: Miyachi et al. describe in this manuscript their experience with large-scale SARS-CoV-2 screening of participants at the Tokyo Olympics, and state that it provides a model for future infectious disease responses to large-scale sporting events. The two-stage strategy proposed and implemented by the authors, using antigen quantification testing prior to PCR, was presumably beneficial, but more information in support of this would be helpful.

**Response**:

Thank you for reviewing our manuscript and for providing comments, which have helped us to improve our manuscript. We have made revisions based on your comments and have provided our responses below. We hope that our responses and revisions adequately address your comments.

The scientific backbone of this manuscript has been described in the previous studies, showing a two-step strategy for detection of SARS-CoV-2 infection comprising screening of self-collected saliva samples using a chemiluminescence enzyme immunoassay (CLEIA) followed by confirmatory testing of saliva and nasopharyngeal swab (NPS) samples using polymerase chain reaction (PCR).

Unfortunately, we are unable to provide additional data on the clinical performance of the tests because there are restrictions on sharing of data on clinical performance other than those contained in press releases from the Tokyo 2020 Organizing Committee, because of a confidentiality agreement.

Nevertheless, we added information as much information as possible. The analytical performance of CLEIA has been shown to have high concondance with the PCR results. We added the cut-off value of CLEIA:

**(Lines 104-106)**

“All specimens were tested by CLEIA with positive and negative thresholds of 4.0 pg/mL and 0.67 pg/mL, respectively, using the specified reagents according to the manufacturer’s instructions.”

**(Lines 196-199)**

“Overall, 11,417 and 4,403 athletes participated in the Olympic and Paralympic Games, respectively. A total of 418,506 screening tests were performed on athletes and team officials during the Games. The screening detected 53 cases, which were later confirmed. The positivity rate was 0.03% (53/15,820).”

**Response to Major comments.**

(1) Page 7, lines 148-150: Although the mean of TAT is given, I feel that this alone makes it difficult to assess the bias of the values. Would it be possible to show the distribution in the raw data or information on quartiles?

**Response**: Thank you for your comment. We have provided the median TAT and the interquartile range:

**(Lines 156-158)**

“The median TAT was 140 minutes (interquartile range: 103–197 minutes), aligning with the target of a mean TAT of 150 minutes for confirmatory tests.”

(2) Page 8, lines 179-182: It seems to me that in order to assess the actual extent of disadvantage due to false-positive results, information such as the results of the second PCR test among those who were positive in the first stage of antigen quantification testing is needed. Although the final percentage of positives is shown in page 10, lines 230-231, I think that these should be included in the Result together with the above breakdown.

**Response**:

Thank you for your suggestion. We mentioned a risk of false positive results for CLEIA tests and our efforts to avoid it, as follows:

**(Lines 174-180)**

“Visual examination of specimens revealed sediments, which caused nonspecific reactions, increasing a risk of false-positive results, rates of re-measurement, and thus prolonging the TAT. We requested the TOCOG to re-educate CLOs about appropriate collection (e.g. prior to eating). To minimize the occurrence of nonspecific reactions due to sediments, the dilution buffer was changed from phosphate buffered saline to a proprietary buffer. As a result, the rate of repeated measurement after re-centrifugation decreased from 8.0% (16/200) to 0.5% (1/200).”

We added the cut-off value of the CLEIA:

**(Lines 104-106)**

“All specimens were tested by CLEIA with positive and negative thresholds of 4.0 pg/mL and 0.67 pg/mL, respectively, using the specified reagents according to the manufacturer’s instructions.”

**(Lines 196-199)**

“Overall, 11,417 and 4,403 athletes participated in the Olympic and Paralympic Games, respectively. A total of 418,506 screening tests were performed on athletes and team officials during the Games. The screening detected 53 cases, which were later confirmed. The positivity rate was 0.03% (53/15,820).”

(3) Page 10, lines 226-227: It is mentioned that the PCR test has a potential risk of false positives, but I think that the risk of false positives for antigen quantification tests should also be mentioned. Rather, to reduce the risk of final false-positives, is the design of antigen quantification as a prior step to the PCR method more important?

**Response**:

Thank you for your suggestion. We have added the following description of the performance of the CLEIA, as you suggested:

**(Lines 63 to 69)**

“Solutions to improve the efficiency of mass screening for SARS-CoV-2 included implementing a two-step screening process comprising screening of self-collected saliva samples using a chemiluminescence enzyme immunoassay (CLEIA), followed by confirmatory PCR testing [3-5]. The scientific framework of this strategy has been reported previously. This strategic allocation of PCR tests to cases that were CLEIA-positive or inconclusive not only maintained accuracy but also significantly improved logistical efficiency in administering mass screening.”

The rationale of the two-step design of antigen quantification prior step to PCR testing was described as follows:

**(Lines 58-66)**

“Although the “gold standard” of SARS-CoV-2 testing by means of polymerase chain reaction (PCR) using nasopharyngeal (NP) swab samples is accurate and reliable, the turnaround time (TAT) for reporting the results may take 24–48 h. Such delays may lead to not only further transmission of disease but can also diminish the daily activity of athletes for training and arrival at the event venues. Furthermore, it requires trained professionals in full protective equipment to collect specimens individually, one person at a time. Solutions to improve the efficiency of mass screening for SARS-CoV-2 included implementing a two-step screening process comprising screening of self-collected saliva samples using a chemiluminescence enzyme immunoassay (CLEIA), followed by confirmatory PCR testing.”

We mentioned a risk of false-positive CLEIA results and our efforts to avoid them, as follows:

**(Lines 174-180)**

“Visual examination of specimens revealed sediments, which caused nonspecific reactions, increasing a risk of false-positive results, rates of re-measurement, and thus prolonging the TAT. We requested the TOCOG to re-educate CLOs about appropriate collection (e.g. prior to eating). To minimize the occurrence of nonspecific reactions due to sediments, the dilution buffer was changed from phosphate buffered saline to a proprietary buffer. As a result, the rate of repeated measurement after re-centrifugation decreased from 8.0% (16/200) to 0.5% (1/200).”

**Response to Minor comments**

(1) Page 3, Line 51: "Tokyo 2020 Games" should be changed to "Tokyo 2020 Olympic and Paralympic Games".

**Response**: Thank you for pointing this out. As you suggested, we changed "Tokyo 2020 Games" to "Tokyo 2020 Olympic and Paralympic Games".

(2) Page 3, Line 63: "instead of PCR screening" alone does not make sense, so the wording should make it clear that it is a comparison with PCR alone.

**Response**: Thank you for your suggestion. To avoid confusion, we deleted the wording "instead of PCR screening alone."

(3) Page 7, lines 167-171: Can you give the information regarding sample number of quality assessment by amylase measurement?

**Response**: Thank you for your question. One hundred samples underwent amylase measurement for quality assurance. We have added this information:

**(Lines 181-184)**

“We evaluated the quality of 100 specimens using amylase measurement with CicaLiquid AMY (Kanto Chemical Co., Inc., Tokyo, Japan) on a fully automated analyzer (JCA-BM8040, JEOL Ltd., Tokyo, Japan). A high proportion of specimens showed amylase levels under the reference range, suggesting inappropriate storage of specimens at ambient temperature.”
